# Leveraging genetic propensity to identify modifiable factors for the age at onset of Alzheimer's disease

**DOI:** 10.1002/alz.71111

**Published:** 2026-02-08

**Authors:** Yi‐Ju Li, Jong Ok La, Adam Naj, Eden R. Martin

**Affiliations:** ^1^ Department of Biostatistics and Bioinformatics Duke University School of Medicine Durham North Carolina USA; ^2^ Duke Molecular Physiology Institute Duke University School of Medicine Durham North Carolina USA; ^3^ Centre for Biomedical Data Science Duke‐NUS Medical School Singapore Singapore; ^4^ Department of Biostatistics Epidemiology, and Informatics University of Pennsylvania Perelman School of Medicine Philadelphia Pennsylvania USA; ^5^ John P. Hussman Institute for Human Genomics University of Miami Miller School of Medicine Miami Florida USA; ^6^ John T. MacDonald Foundation Department of Human Genetics University of Miami, Miller School of Medicine Miami Florida USA

**Keywords:** age at onset, causal association, linear mixed model, polygenic score, survival model

## Abstract

**INTRODUCTION:**

Knowledge of modifiable factors influencing age at onset (AAO) of Alzheimer's disease (AD) remains limited. This study utilizes genetic information to uncover such factors.

**METHODS:**

Using 43 exposure genome‐wide association studies (GWAS) summary statistics, we calculated corresponding polygenic scores (PGS) for 9219 AD cases and 10,345 controls from the Alzheimer's Disease Genetic Consortium (ADGC). Linear mixed model and survival analyses were performed to identify exposure‐PGS associated with AAO. Top exposures were cross‐evaluated using PGS from the PGS Catalog and Mendelian randomization (MR) for causal relationships.

**RESULTS:**

Eight exposures showed significant exposure‐PGS associations with AAO of AD. Higher educational attainment, better cognitive performance, and greater relative fat intake were associated with later AAO; whereas the remaining were linked to earlier onset. MR analysis indicated a causal relationship between AAO and educational attainment, cardiovascular disease, and type 2 diabetes (T2D).

**DISCUSSION:**

The eight modifiable factors, particularly educational attainment, cardiovascular disease, and T2D, may facilitate early intervention to delay the onset of AD.

**Highlights:**

- We screened 43 modifiable factors for their association with the age at onset (AAO) of Alzheimer's disease (AD) using polygenic scores (PGS) as the proxy for the exposure.
- Higher educational attainment, better cognitive performance, and greater relative fat intake were linked to later AAO, suggesting an enhanced resilience against AD.
- Type 2 diabetes, cardiovascular disease, major coronary heart disease, and increased low‐density lipoprotein (LDL) ‐cholesterol and total cholesterol are associated with earlier AAO.
- Mendelian randomization analysis revealed causal effects of educational attainment, type 2 diabetes, and cardiovascular disease on AAO of AD.

## 1 BACKGROUND

Alzheimer's disease (AD) is the leading cause of dementia among older adults, predominantly occurring in individuals aged 65 and older. AD is characterized by progressive cognitive decline resulting from neuronal damage in brain areas that govern memory, language, and thinking.^1^ In the United States, the prevalence of AD increases with age: it affects 5% of individuals aged 65–74, 13.1% of those aged 75–84, and 33.3% of those aged 85 and above.^1^ Delaying the age at onset (AAO) of AD could help reduce prevalence rates among younger age groups, an alternative solution to enhancing resilience against AD.

Beyond aging, genetics, and family history, several modifiable risk factors have been linked to AD. These factors (hereafter referred to as ‘exposures’) can be categorized into education, psychosocial elements, comorbidities, lifestyles, and other exposures.^2^ Various population‐specific Dementia Risk Scores (DRS),^3^, ^4^, ^5^, ^6^ such as the UK Biobank Dementia Risk Score (UKBDRS),^6^ have been developed by incorporating multiple modifiable factors to predict the risk of all‐cause dementia. To date, most identified modifiable factors have been associated with the risk of developing AD.^2^ It remains unclear whether any of them influence the AAO of AD.

Data availability is a key challenge in studying modifiable factors for AAO of AD. While electronic health records may capture various clinical factors, the AAO of AD patients may not be fully documented or reliably verified. Conversely, AD research datasets, such as those cohorts from the Alzheimer's Disease Genetic Consortium (ADGC), typically offer well‐curated AAO data, but most lack comprehensive information on exposure factors. Integrating genetic information through approaches like polygenic scores (PGS) and Mendelian randomization (MR) to infer exposures and their relationship with outcomes offers an alternative strategy to address this challenge.

Many summary statistics from genome‐wide association studies (GWAS) are now available. PGS refers to the sum of weighted genotype scores from an optimal set of genetic variants that explain the most trait variance, analogous to an individual's genetic propensity for the trait. For disease outcomes, a robust PGS can serve as a useful tool for identifying individuals at increased risk of developing the disease. PGS can be applied to a cross‐trait analysis, where PGS for an exposure can serve as the proxy of that exposure for the association analysis with a trait of interest.^7^ Andrews et al. (2021) performed PGS cross‐trait analysis using 22 exposure‐GWAS summary statistics to test their PGS association with AD risk.^8^ Higher educational attainment and lower diastolic blood pressure were significantly associated with reduced AD risk. However, they did not examine the relationship between exposures and AAO of AD.

MR is another method that leverages genetic variants as instrumental variables (IVs) to infer the causal relationship between factors and outcomes. MR shares a similar concept to randomized control trials (RCTs), based on the principle that genetic variants are randomly assorted at conception, providing a natural form of randomization. Using the two‐sample MR analyses, Andrews et al.^8^ identified that higher educational attainment has a causal effect on reducing AD risk and delaying AAO. They also showed that type 2 diabetes (T2D) has a causal association with earlier AAO but no causal effects on overall AD risk.

With more PGS studies published, the PGS Catalog serves as a repository for PGS published to date for a wide range of traits. Alternatively, exposure‐PGS can be directly computed for the target dataset by using the effect sizes (i.e., variant weights) from the corresponding exposure‐PGS entries in the PGS Catalog. In this study, we aimed to: (1) identify modifiable factors that influence the AAO of AD using PGS; (2) compare the performance between two types of PGS–those constructed from exposure‐GWAS summary statistics, and those directly calculated based on published exposure‐PGS from the PGS Catalog; and (3) assess the causal relationship between the top associated factors and AAO of AD through MR analysis.

## 2 METHODS

### 2.1 Outcome and AD genetic data

**FIGURE 1 alz71111-fig-0001:**
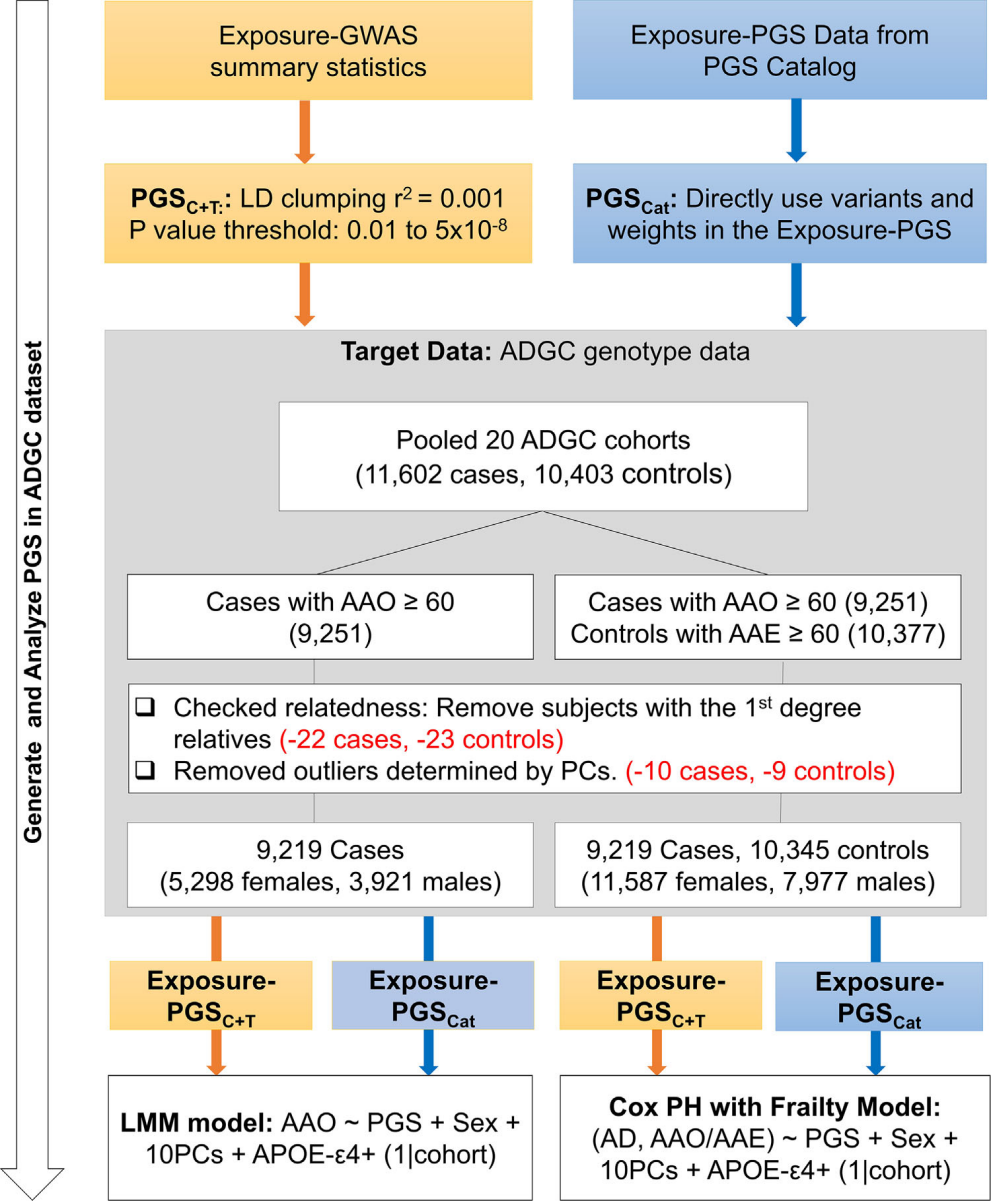
Overview of the analysis workflow and CONSORT diagram for the ADGC genetic dataset. Two sets of PGS for exposures were generated: (1) PGS_C+T_, using the clumping and *p*‐value thresholding method based on exposure GWAS summary statistics; and (2) PGS_Cat_, derived from effect sizes of variants included in the published PGS in the PGS Catalog. Associations between PGS and AAO of AD were assessed using two approaches: a LMM applied to cases only, and a CoxPHF incorporating both cases and controls. AAO, age at onset; AD, Alzheimer's disease; ADGC, Alzheimer's Disease Genetic Consortium; CoxPHF, Cox proportional hazards model with frailty; GWAS, genome‐wide association studies; LMM, linear mixed model; PGS, polygenic scores.

The same AD genetic dataset previously used in our GWAS study for the AAO of AD^9^ was analyzed. This dataset consists of pooled genome‐wide imputed genotype data from 20 cohorts of the ADGC. The AAO of AD refers to the age at which AD‐related symptoms first appear, reported by the individuals or their informants. For a small portion (∼15%) of the individuals without the AAO information, age at ascertainment was used instead.^10^ For unaffected individuals (controls), we used the last known age at exam (AAE) as the censoring age. After excluding the related subjects and those with missing age information or age below 60, the analysis dataset comprises 9219 AD cases with AAO and 10,345 controls with AAE (Figure 1). All are of European ancestry.

**RESEARCH IN CONTEXT**

1. **Systematic review**: The authors reviewed literature using PubMed and meeting abstracts and presentations. To date, most studies for modifiable factors have been for Alzheimer's disease (AD) risk. There are limited studies that reported modifiable factors influencing the age at onset (AAO) of AD.
2. **Interpretation**: Using polygenic scores (PGS) as genetic proxies for exposure factors, we screened 43 modifiable factors for associations with the AAO of AD. Eight exposures were identified, with consistent results across analyses. Notably, higher educational attainment, better cognitive performance, and increased relative fat intake were linked to later AAO, while others were associated with earlier AAO. Educational attainment, cardiovascular disease, and type 2 diabetes, factors with the strongest association, also demonstrated causal effects on the AAO of AD.
3. **Future directions**: Future research should replicate these factors and develop integrated prediction models combining genetic and modifiable exposures to enable personalized strategies aimed at enhancing cognitive resilience to delay the onset of AD.

### 2.2 Exposures and GWAS summary statistics

We compiled 42 modifiable factors (i.e., exposures), including cardiovascular disease risk, pre‐existing comorbidities, and environmental, lifestyle, and metabolic factors, and examined their relationship with the AAO of AD (Table 1). GWAS summary statistics were obtained from large biobanks (e.g., UK Biobank (UKB) and FinnGen) and consortia. We prioritized GWAS studies with publications. If no publications are[Fig alz71111-fig-0001] available, we choose the ones with the largest sample size. GWAS summary statistics were downloaded from the Integrative Epidemiology Unit (IEU) OpenGWAS Project database or the original result repository described in the respective publications. These exposure‐GWAS summary statistics consist of beta or odds ratio (OR) estimates and *p*‐values of genome‐wide variants. Using the GWAS summary statistics of three fatty acid components, polyunsaturated, monounsaturated, and saturated fatty acid levels for meta‐analysis, we generated GWAS summary statistics for total fatty acid levels as a proxy of high‐fat intake exposure. That is, we performed multi‐trait analysis of GWAS (MTAG)^11^ of these three traits first, followed by a meta‐analysis using METAL.^12^ This led to a total of 43 exposures investigated in this study, with sample sizes of exposure‐GWAS summary statistics ranging from 83,726 to 1,597,374 (Table 1).

**TABLE 1:** List of 43 modifiable factors (exposures) and the respective source of GWAS summary statistics.

		GWAS summary statistics		
Category	Trait	Study	*N*	CONSORTIUM
Cardiovascular disease	Atrial fibrillation	Roselli et al. (2018)^13^	537,409	AFGen, Broad AF, UK Biobank, BBJ
	Cardiovascular disease	Loh et al. (2018)^14^	477,807	UKB
	Heart failure	Shah et al.(2020) ^15^	977,323	HERMES
	Hypertension		463,010	MRC‐IEU
	Major coronary heart disease event		361,194	UKB
	Peripheral artery disease	Sakaue et al. (2021)^16^	483,078	BBJ, UKB, FinnGen
	Ventricular arrhythmia	Sakaue et al. (2021)^16^	328,216	BBJ, UKB, FinnGen
Environmental	Air pollution: nitrogen dioxide		456,380	MRC‐IEU
	Air pollution: nitrogen oxides		456,380	MRC‐IEU
	Air pollution: particulate matter (pm10)		423,796	MRC‐IEU
	Air pollution: particulate matter (pm2.5)		423,796	MRC‐IEU
Lifestyle	Age of smoking initiation	Saunders et al. (2022) ^17^	323,386	GSCAN
	Cigarettes per day	Saunders et al. (2022) ^17^	326,497	GSCAN
	Diet: relative carbohydrate intake	Meddens et al. (2021)^18^	268,922	UKB; DietGen
	Diet: relative fat intake	Meddens et al. (2021)^18^	268,922	UKB; DietGen
	Diet: relative protein intake	Meddens et al. (2021)^18^	268,922	UKB; DietGen
	Diet: relative sugar intake	Meddens et al. (2021)^18^	235,391	UKB; DietGen
	Drinks per week	Saunders et al. (2022) ^17^	666,978	GSCAN
	Insomnia self reported	Lane et al. (2019)^19^	453,379	UKB
	Moderate‐vigorous physical activity	Klimentidis et al. (2018)^20^	377,234	UKB
	Sleep duration, self‐reported	Lane et al. (2019)^19^	83,726	UKB
	Sleep apnea syndrome	Sakaue et al. (2021)^16^	476,853	BBJ, UKB, FinnGen
	Smoking behavior	Saunders et al. (2022) ^17^	805,431	GSCAN
Metabolic	BMI	Yengo et al. (2022)^21^	1,597,374	GIANT
	Diastolic blood pressure	Surendran et al. (2020)^22^	810,865	UKB
	High‐density lipoprotein cholesterol (HDL‐cholesterol)	Liu et al. (2017)^23^	316,391	GLGC
	Low‐density lipoprotein cholesterol (LDL‐cholesterol)	Liu et al. (2017) ^23^	295,826	GLGC
	Monounsaturated fatty acid levels	Richardson et al. (2022)^24^	115,006	UKB
	Polyunsaturated fatty acids levels	Richardson et al. (2022)^24^	115,006	UKB
	Saturated fatty acid levels	Richardson et al. (2022)^24^	115,006	UKB
	Total fatty acid levels	Meta‐analysis of the above three fatty acid levels	115,006	UKB
	Systolic blood pressure	Surendran et al. (2020)^17^	810,865	UKB
	Total cholesterol	Liu et al. (2017)^23^	319,677	GLGC
	Triglycerides	Liu et al. (2017)^23^	305,699	GLGC
Pre‐existing conditions	Age‐related hearing impairment	Kalra et al. (2020)^25^	330,759	UKB
	Cognitive performance	Lee et al. (2018)^26^	257,828	SSGAC
	Depression	Howard et al. (2019)^27^	500,199	UKBB; PGC; deCODE; iPSYCH; GeneScotland; GERA
	Educational attainment	Okbay et al. (2022)^28^	765,283	SSGAC
	Head injury	Sakaue et al. (2021)^16^	357,658	BBJ, UKB, FinnGen
	Mood swing		451,619	MRC‐IEU
	Neurotic, stress‐related and somatoform disorders		218,792	FinnGen
	Social isolation	Day et al. (2018)^29^	445,024	UKB
	Type 2 diabetes	Xue et al. (2018)^30^	605,056	DIAGRAM; UKBB; GERA

Abbreviations: BMI, body mass index; GWAS, genome‐wide association studies;

### 2.3 PGS constructed from GWAS summary statistics

Linkage disequilibrium (LD) clumping and *p*‐value threshold (C+T) were used to construct exposure‐PGS by utilizing variant effect size estimates (i.e., $\hat{\beta }$) from the exposure GWAS summary statistics (exposure‐GWAS) (Figure 1). First, we filtered variants with *p*‐values ≤0.01 from each exposure‐GWAS summary statistic to ensure that the variants included in the PGS were potentially associated with the exposure. Second, LD clumping was performed based on *r* ^2^ ≤0.001 within a 10 MB window to assemble a set of independent variants.^8^ Third, variants were selected using different *p*‐value thresholds (0.01, 0.001, 10^−^^4^, 10^−^^5^, 10^−^^6^, 10^−^^7^, 5 × 10^−^^8^) from exposure‐GWAS summary statistics to calculate PGS for each individual in the AD genetic dataset (the target dataset), following the formula, $PGS=\sum_{i}^{k}\hat{\beta_{i}}G_{i}$, where $G_{i}$ is the genotype score of variant *i*. Finally, we compared model performance across PGS derived from different p‐value thresholds to determine the optimal set of variants for PGS. Similar to our previous AAO GWAS,^9^ we applied two statistical models. One is the case‐only analysis, where the AAO of AD cases was treated as a quantitative trait. The linear regression model was employed to regress AAO on PGS, and then the optimal PGS was determined based on the highest *R* ^2^ value. The other is the survival analysis, where AD was treated as a survival outcome with time to AD event observed at AAO for AD cases and censored at AAE for controls. The Cox proportional hazard (CoxPH) model was applied to evaluate the PGS effect on the hazard of an AD event at a given time. The optimal PGS was determined based on the highest concordance index (C‐index). *PRSice 2.0* and *coxph()* function in R Version 4.4.2 were used as appropriate.

For each optimal exposure‐PGS generated based on the C+T approach (referred to as *PGS_C+T_*), we assessed its association with the AAO of AD using a fully adjusted model. The *PGS_C+T_* was first scaled to a *Z*‐score. For the case‐only analysis, the linear mixed models (LMMs) was used to regress AAO on *PGS_C+T_*, sex, apolipoprotein E (APOE) ‐ε4 dosage, and the top 10 principal components (PCs) with a random intercept by cohorts for each exposure. The PCs were constructed based on genome‐wide independent variants as previously described.^9^ For survival analysis, the Cox proportional hazard with frailty (CoxPHF) model was employed to model the hazard function of AD on *PGS_C+T_* and the same covariates with a random intercept by cohorts for each exposure. For each survival analysis, we also tested the proportional hazard (PH) assumption, that is, the hazard ratios (HR) remain constant over time, using the *cox.zph() R* function, and visually inspected Schoenfeld residuals plotted against event time for main covariates (PGS, age, and sex). For exposures with covariates showing residuals systematically deviated from the horizontal line, we included a time–covariate interaction term in the CoxPHF model. Finally, exposure was considered significant if the corresponding *PGS_C+T_* achieves *p* < 0.001, a Bonferroni‐corrected significance threshold for 43 exposures.

### 2.4 PGS derived from the PGS catalog

The PGS Catalog is a repository for published PGS, each trained to optimize prediction for its corresponding trait. As of March 2025, 5061 PGS for 657 traits from 700 publications are available. Each PGS consists of variant weights (i.e., beta or odds ratio estimates) for variants included in the PGS. For exposures where *PGS_C+T_* achieved nominal significance (*p* < 0.05) from the analysis above, we downloaded the available corresponding PGS from the PGS Catalog. The variant weights were directly applied to compute the corresponding exposure‐PGS (hereafter referred to as *PGS_Cat_)*. *PGS_Cat_* was standardized as a *Z*‐score and tested using the same fully adjusted LMM and CoxPHF to assess its association with the AAO of AD. To compare PGS_Cat_ and PGS_C+T_, we computed the Pearson correlation between these two PGS and examined AAO distributions across quartiles of each PGS.

### 2.5 Delineate the relationship between exposures

For the exposures showing a significant association with AAO, we further investigate whether they are independent predictors. Pairwise Pearson correlation between exposure‐PGSs was computed. For those with an absolute correlation coefficient (*r*) > 0.2, we modeled them jointly on the LMM and CoxPHF with the same covariate adjustments to examine their dependency in influencing the AAO of AD.

### 2.6 MR analysis

The top significant exposures identified from the PGS analyses were further evaluated for their causal relationship with the AAO of AD by MR analysis. We primarily conducted two‐sample MR analyses, incorporating GWAS summary statistics for both the exposure and AAO of AD.^9^ For validation, we also performed one‐sample MR using the external variant weights for the exposure. MR requires IVs that adhere to the following assumptions: (1) IVs are associated with the exposure of interest; (2) IVs are not associated with any confounders of the exposure‐outcome association; and (3) IVs are not directly associated with the outcome.

Selection of IVs were based on the following criteria for each exposure: (1) variants not in LD (*r* ^2^ ≤ 0.001); (2) MAF ≥0.05; (3) variants meeting *p* < 5 × 10^−8^ and an *F* statistic >10 from exposure‐GWAS summary statistics; and (4) exclusion of variants with *p* < 5 × 10^−6^ from outcome‐GWAS summary statistics and all variants in the APOE region to ensure no association with the AAO of AD. Since GWAS summary statistics from survival analysis would lead to biased MR results,^31^ MR was only performed based on the GWAS summary statistics from the case‐only LMM analysis for AAO of AD we previously published.^9^ The same MR analysis was performed by relaxing the selection of IVs to variants meeting *p* < 5 × 10^−6^ as a sensitivity analysis to match the threshold used in Andrews et al.^8^

The effect sizes of exposure and outcome GWAS summary statistics were harmonized to match the effect allele (A1). The inverse variance weighted (IVW) ^32^ was the primary method to test the causal effect of the exposure on outcome, employing the *TwoSampleMR* R package. Sensitivity analyses were performed to assess pleiotropy and the robustness of the causal effect. These included the weighted median^33^ and weighted mode^34^ methods, which provide estimates robust to horizontal pleiotropy and potential invalid IVs, respectively, the MR‐Egger^35^ and heterogeneity tests to assess horizontal pleiotropy effects and the validity of IVs, and the MR‐PRESSO to detect horizontal pleiotropy and distortion of causal effects due to outliers.^36^ We also examined reverse causality for the significant exposures by changing the direction of two‐sample MR analysis from AAO to the exposure. Finally, we performed negative control MR analyses to examine the validity of our MR analysis procedure using height and rheumatologic condition as the exposures.

One‐sample MR was performed using a two‐stage least squares (2SLS) framework, incorporating external variant weights to generate genetically predicted exposure based on the strong IV criteria outlined above.^37^, ^38^ The analysis was similar to that for PGS_C+T_ except that variants included were those meeting *p* < 5 × 10^−8^ and 5 × 10^−6^, respectively.

## 3 RESULTS

The information on GWAS summary statistics for 43 exposure factors is listed in Table 1. The AD dataset, served as the target dataset, includes 9,219 AD cases and 10,345 controls pooled from 20 ADGC cohorts (Figure 1). There is a higher proportion of females (59.2%), and as expected, the frequency of APOE‐ε4 was greater in AD cases than in controls (0.34 vs. 0.12).

### 3.1 Exposure‐PGS association with AAO

The analysis using PGS_C+T_ identified six exposures significantly associated with the AAO of AD with *p* < 0.001, a Bonferroni‐adjusted threshold (Table 2). The full results for 43 exposures are listed in Table S1. T2D and sleep apnea showed violation of the PH assumption on APOE‐ε4 and sex (Figure S1). Therefore, CoxPHF incorporated time×APOE‐ε4 and time×sex interaction terms for these exposures. Notably, all six significant exposure‐PGS_C+T_ demonstrated consistent directional effects on AAO in both case‐only (LMM) and survival analyses (CoxPHF). For example, a negative beta from LMM and HR >1 from CoxPHF imply an association with a younger AAO of AD. Among them, educational attainment is the most significant exposure for AAO. Specifically, each 1 standard deviation (SD) increase in the PGS_C+T_ score for educational attainment could delay an average of 0.38 years in AAO of AD (beta (95% confidence interval [CI]) = 0.38 (0.24, 0.52), *p* = 7.8 × 10^−8^; HR (95% CI) = 0.92 (0.9, 0.94), *p* = 1.77 × 10^−14^. As expected, better cognitive performance is associated with delayed AAO of AD (beta (95% CI) = 0.27 (0.13, 0.4) and HR (95% CI) = 0.93 (0.91, 0.95). Conversely, T2D, cardiovascular disease, and smoking behavior are associated with an earlier AAO of AD. Finally, relative fat intake reached significance in the survival analysis (HR (95% CI) = 0.95 (0.93, 0.98), *p* = 3.46 × 10^−5^) and borderline significance in the LMM analysis (beta(95% CI) = 0.24 (0.09, 0.39), *p* = 0.002). In contrast, PGS for individual saturated, unsaturated, and total fatty acid components did not reach statistical significance, except LMM analysis of PGS for total fatty acid levels showed potential association with AAO of AD (beta (95% CI) = ‐0.16 (‐0.29, ‐0.02), *p* = 0.028) (Table S1).

**TABLE 2:** Top exposures associated with AAO of AD based on PGS_C+T_ and PGS_Cat_.

	PGS_C + T_				PGS_Cat_			
	LMM		CoxPHF		LMM		CoxPHF	
Exposure	Beta (95% CI)	*p*‐value	HR (95% CI)	*p*‐value	Beta (95% CI)	*p*‐value	HR (95% CI)	*p*‐value
***p* < 0.001 from PGS_C+T_**								
Educational attainment	0.38 (0.24, 0.52)	7.80×10^−8^	0.92 (0.9, 0.94)	1.77×10^−14^	0.37 (0.23, 0.51)	1.35×10^−7^	0.92 (0.9, 0.94)	1.33×10^−14^
Type 2 diabetes	−0.32 (−0.46, −0.18)	4.85×10^−6^	1.04 (1.02, 1.06)	2.76×10^−4^ ^a)^	−0.24 (−0.38, ‐0.1)	6.35×10^−4^	1.02 (1, 1.05)	0.023
Cardiovascular disease	−0.31 (−0.44, −0.17)	1.21×10^−5^	1.04 (1.02, 1.06)	1.78×10^−4^	−0.38 (−0.52, −0.24)	6.58×10^−8^	1.07 (1.05, 1.09)	5.32×10^−10^
Smoking behavior	−0.27 (−0.4, −0.13)	1.56×10^−4^	1.05 (1.03, 1.08)	5.46×10^−7^	−0.02 (−0.16, 0.11)	0.731	0.99 (0.97, 1.01)	0.355
Cognitive performance	0.27 (0.13, 0.4)	1.49×10^−4^	0.93 (0.91, 0.95)	6.58×10^−13^	–	–	–	–
Diet: relative fat intake	0.24 (0.09, 0.39)	0.002	0.95 (0.93, 0.98)	3.46×10^−5^	–	–	–	–
**0.001 ≤ *p* < 0.05 from PGS_C+T_ and *p* < 0.0035 from PGS_Cat_**								
Major coronary heart disease event	−0.19 (−0.33, −0.05)	0.006	1 (0.98, 1.02)	0.906	−0.33 (−0.47, −0.19)	3.07×10^−6^	1.05 (1.03, 1.07)	1.21×10^−5^
low‐density lipoprotein cholesterol (LDL‐cholesterol)	−0.19 (−0.34, −0.05)	0.010	1.02 (1, 1.04)	0.075	−0.22 (−0.36, −0.07)	0.003	1.04 (1.02, 1.06)	8.33×10^−4^
Total cholesterol	−0.18 (−0.32, −0.04)	0.015	1.02 (1, 1.05)	0.031	−0.21 (−0.35, −0.06)	0.005	1.04 (1.01, 1.06)	0.001
Age of smoking initiation	0.13 (0, 0.27)	0.055	0.97 (0.95, 0.99)	0.003	−0.17 (−0.31, −0.03)	0.018	1.04 (1.01, 1.06)	0.001
BMI	−0.02 (−0.16, 0.12)	0.767	0.97 (0.95, 0.99)	0.004	−0.37 (−0.51, −0.24)	1.00×10^−7^	1.05 (1.03, 1.07)	1.82×10^−5^ ^a)^

*Note*: For cognitive performance and relative fat intake, PGS were not available in the PGS Catalog.

Abbreviation: BMI, body mass index; CI, confidence interval; CoxPHF, Cox proportional hazard model with frailty to account for cohort random effect; HR, hazard ratio; LD, linkage disequilibrium; LMM, linear mixed model; PGS_C+T_, PGS constructed based on LD clumping and *p*‐value threshold by PRSice‐2; PGS_Cat_, PGS calculated based on the variants and the corresponding effect sizes from the PGS Catalog.

^a)^ CoxPHF model for T2D (PGS_C+T_) and BMI (PGS_Cat_) included additional adjustment of time×APOE‐ε4 and time×sex interaction terms to address the violation of the PH assumption detected for both covariates.

Among 27 exposures meeting PGS_C+T_ < 0.05, 13 exposures have published PGS available from the PGS Catalog for generating PGS_Cat_ in our AD genetic dataset (Tables S1 and S2). Since body mass index (BMI) showed violation with the PH assumption on APOE‐ε4 and sex (Figure S2), similarly, the CoxPHF model adjusted for additional interaction terms. Eight exposure‐PGS_Cat_ achieved significance threshold *p* < 0.0035 in either LMM or CoxPHF analysis (Table 2). Educational attainment, cardiovascular disease, and type 2 diabetes were validated, consistent with PGS_C+T_ (top portion of Table 2). Additionally, major coronary heart disease events, low‐density lipoprotein cholesterol (LDL‐cholesterol), and total cholesterol were identified by the PGS_Cat_ approach, though not significant in the PGS_C+T_ analyses, the directional effects were consistent (bottom portion of Table 2). However, smoking behavior was not validated by PGS_Cat_ (*p* = 0.731 for LMM, 0.355 for CoxPHF), and age of smoking initiation and BMI showed different directional effects from PGS_C+T_ and between LMM and CoxPHF. Therefore, these three factors should be ruled out.

Pairwise correlations of the remaining eight top exposures in Table 2 showed low‐moderate correlations (*r* = 0.22) between educational attainment and cognitive performance and high correlation (*r* = 0.84) between LDL and total cholesterol (Table S3a). Jointly modeling these pairs of exposures showed that educational attainment and cognitive performance are independent predictors for AAO of AD, implying their independent effects on AAO of AD. The joint modeling of LDL cholesterol and total cholesterol resulted in diminished association signals due to the strong collinearity (Table S3b), implying both cholesterol measures shared the same association signals on AAO of AD.

### 3.2 Comparison between exposure‐PGS_C+T_ and ‐PGS_Cat_

**FIGURE 2:**
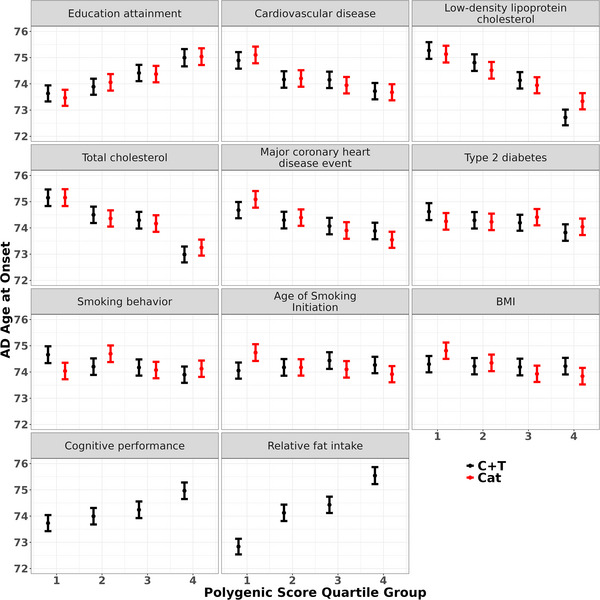
Mean AAO of AD and their 95% confidence intervals are shown for each PGS quartile for the 11 exposures listed in Table 2. Both PGS_C+T_ (black) and PGS_Cat_ (red) are presented. For relative fat intake and cognitive performance, PGS data were not available in the PGS Catalog; therefore, only PGS_C+T_ are shown for these exposures. AAO, age at onset; AD, Alzheimer's disease; PGS, polygenic scores.

For the nine exposures with both types of PGS data in Table 2, significant correlations were observed between PGS_C+T_ and PGS_Cat_ for most of them (Table S4). Notably, educational attainment, cardiovascular disease, LDL‐cholesterol, and total cholesterol showed the highest correlation between PGS_C+T_ and PGS_Cat_, with Pearson coefficients ranging from 0.429 to 0.511. Figure 2 illustrates the distribution of AAO by quartiles of PGS for all 11 top exposures (Table 2). Overall, the AAO distributions by Exposure‐PGS quartiles were similar between PGS_C+T_ and PGS_Cat_. More AAO difference between the 1^st^ and the 4^th^ quartiles of PGS was observed in all exposures except T2D, smoking behavior, age of smoking initiation, and BMI. For instance, individuals in the 4^th^ quartile of educational attainment PGS experienced an average of AAO 1.4 to 1.6 years later than those in the 1^st^ quartile (mean AAO: 75.0 vs. 73.6 for PGS_C+T_ and 75.0 vs, 73.5 for PGS_Cat_, both *p* < 0.001). Interestingly, the delay of AAO is even larger between the 4^th^ and 1^st^ quartiles for LDL‐cholesterol (delayed 2.6 years for PGS_C+T_; 1.8 years for PGS_Cat_) and relative fat intake (2.7 years for PGS_C+T_).

### 3.3 Exposures with potential causal association with the AAO of AD

We further investigated the 11 exposures in Table 2 for a causal association with AAO by performing MR analysis. The same exposure‐GWAS summary statistics (Table 1) were used to select IVs. Both educational attainment (*p* = 2.85 × 10^−5^) and cardiovascular disease (*p* = 0.001) showed significant causal association with AAO by the IVW method (Tables 3 and S4). As expected, reverse causality for AAO of AD influencing these two exposures was not observed (Table S5). T2D did not reach the significance threshold *p* < 0.0045 but showed nominal significance across all MR methods, IVW (p = 0.046), MR EGGER (*p* = 0.026), weighted median (*p* = 0.008), and weighted mode (*p* = 0.026) (Table S4). However, using a conservative IV selection criterion *p* < 5 × 10^−6^, T2D and smoking behavior reached significance (IVW *p* = 0.003, Table S6) along with cognitive performance (*p* = 0.005), LDL‐cholesterol (*p* = 0.015), and total cholesterol (*p* = 0.036) meeting nominal significance. This suggests T2D is likely to have a causal association with AAO of AD. No significant horizontal pleiotropy and outliers were observed for all exposures tested, as shown by the MR‐EGGER intercept and MR‐PRESSO (Tables S5 and S7). One‐sample MR also supports the causal effect findings of educational attainment, cardiovascular disease, and T2D from two‐sample MR (Table S8). Finally, using height and rheumatologic condition as the negative controls, we did not observe a significant causal association with the AAO of AD (*p* = 0.34 and 0.43), which supports the validity of our MR analysis procedure.

**TABLE 3:** Results from Mendelian randomization analysis for the top 11 exposures using IVs meeting *p* < 5×10^−8^ from the exposure‐GWAS summary statistics.

		IVW		Heterogeneity (Q)	Weighted median	Weighted Mode	MR‐Egger	MR‐Egger intercept	MR‐PRESSO global test
Exposure	SNPs	*b* (se)	*p‐Value*	*p‐Value*	*b* (se)	*b* (se)	*b* (se)	*p‐Value*	*p‐Value*
Educational attainment	559	1.48 (0.35)	2.85×10^−5^	0.41	1.35 (0.55)	1.56 (1.45)	1.78 (1.69)	0.95	0.41
Cardiovascular disease	158	−3.75 (1.17)	0.001	0.27	−3.42 (1.74)	0.34 (3.6)	−1.29 (3.74)	0.23	0.26
Low‐density lipoprotein cholesterol	85	−0.62 (0.32)	0.051	0.26	−0.38 (0.48)	−0.08 (0.5)	−0.37 (0.42)	0.17	0.26
Total cholesterol	105	−0.53 (0.29)	0.071	0.46	−0.41 (0.47)	−0.45 (0.5)	−0.47 (0.44)	0.85	0.46
Major coronary heart disease event	6	−0.87 (14.48)	0.95	0.81	−6.72 (17.6)	−25.1 (32.23)	−16.16 (22.12)	0.45	0.79
Type 2 diabetes	130	−0.29 (0.14)	0.046	0.89	−0.67 (0.25)	−0.77 (0.34)	−0.71 (0.31)	0.12	0.89
Smoking behavior	293	−0.35 (0.57)	0.54	0.14	−0.56 (0.86)	1.77 (2.49)	−1.39 (2.32)	0.38	0.13
Age of smoking initiation	11	−1.58 (1.98)	0.43	0.41	−2.32 (2.61)	−13.59 (8.76)	−3.33 (4.14)	0.19	0.43
BMI	2588	0.04 (0.15)	0.8	0.17	−0.06 (0.25)	−0.16 (0.31)	0.16 (0.48)	0.45	0.17
Cognitive performance	158	0.65 (0.42)	0.12	0.9	0.49 (0.63)	−1.07 (2.12)	−0.1 (1.62)	0.41	0.9
Diet: relative fat intake	4	−1.01 (2.3)	0.66	0.53	−0.58 (2.79)	−5.68 (10.04)	−0.21 (3.47)	0.68	0.59

Abbreviations: BMI, body mass index; GWAS, genome‐wide association studies; IV, instrumental variables; IVW, inverse variance weighted; MR, Mendelian randomization; SNP, single‐nucleotide polymorphism.

## 4 DISCUSSION

Utilizing GWAS summary statistics and published PGS to generate genetically predicted exposure factors as proxies, we evaluated 43 modifiable factors for their relationship with the AAO of AD. In this study, we employed two distinct PGS construction methods for exposures and two statistical models for testing the association between exposure‐PGS and AAO. Of the 11 exposures identified by at least one analysis (Table 2), eight demonstrated consistent results with the same directional effects on the AAO across analyses. Specifically, higher educational attainment, better cognitive performance, and greater relative fat intake were associated with a later AAO. In contrast, T2D, cardiovascular disease, major coronary heart disease, elevated LDL‐cholesterol, and higher total cholesterol are associated with earlier AAO. The most promising findings were educational attainment, T2D, and cardiovascular disease, which were consistently detected by both PGS_C+T_ (*p* < 0.001) and PGS_Cat_ (*p* < 0.0035) and subsequently by the MR analysis, implying their causal effects on the AAO of AD. These findings suggest that improving educational attainment and preventing cardiovascular disease and T2D may enhance cognitive resilience and delay the onset of AD.

Educational attainment and T2D have been linked to AD risk by observational studies^2^, ^39^. Using the PGS approach, Andrews et al.^8^ found educational attainment associated with AD risk but not T2D. Our study is likely the first to demonstrate their relationship with AAO of AD. Furthermore, the causal association of educational attainment and T2D with AAO of AD was previously identified by Andrews et al.^8^ through MR analysis using a conservative IV selection criterion *p* < 5 × 10^−6^. Our two‐sample and one‐sample MR analyses revealed consistently significant results for these two exposures based on the same IV selection threshold. Under a more stringent IV selection threshold (*p* < 5 × 10^−8^), educational attainment remained significant. However, T2D still showed nominally significant (IVW *p* = 0.046, MR‐EGGER *p* = 0.026). Overall, these findings from PGS and MR analyses strongly support the role of educational attainment and T2D in influencing the AAO of AD.

Our finding of the causal association between cardiovascular disease and AAO of AD is novel. Andrews et al.^8^ did not test the causal effect of cardiovascular disease on AD risk and AAO, but they evaluated several cardiovascular‐related factors, like blood pressure, high‐density lipoprotein (HDL), LDL, and triglycerides. None of them showed a causal association with AD risk and its AAO. In contrast, our study identified PGS of LDL‐cholesterol and total cholesterol associated with the AAO of AD (Table 2), with marginal evidence for a causal association with the AAO of AD when a conservative IV selection threshold (*p* < 5 × 10^−6^) was applied to both two‐sample and one‐sample MR analysis (Table S6). Additionally, high correlation was observed between these two cholesterol measures (*r* = 0.84) but not with cardiovascular disease (*r* = 0.07). These results suggest that overall cardiovascular health has a stronger impact on the AAO of AD than a specific cardiovascular condition. We also found low correlation between PGSs of cardiovascular diseases and T2D (*r* = 0.08), suggesting that cardiovascular disease and T2D independently influence AAO of AD.

Various studies have investigated the relationship between dietary fat intake and the risk of developing AD, but the results were inconsistent^40^, ^41^, ^42^. Our finding of higher relative fat intake in delaying AAO of AD is novel and may serve as a beneficial intervention target. The original GWAS summary statistics from Meddens et al.^18^ defined relative fat intake as the proportion of energy from the fat intake over the corrected total energy intake, where the correction factor (or called shrinkage factor) for the total energy is the beta coefficient estimated from log(fat intake) regressing on the log(total energy), a log‐log linear model. This relative fat intake measurement did not differentiate between saturated and non‐saturated fat, a potential proxy for high fat intake. However, PGS of individual components of saturated and unsaturated (monounsaturated, polyunsaturated) fatty acid levels did not show an association with AAO of AD. While PGS for total fatty acid levels showed a borderline association with AAO of AD by the LMM analysis (*β* = ‐0.16, *p* = 0.028) with an inverse directional effect, it was not validated by the survival analysis. Therefore, our finding of higher relative fat intake associated with later AAO of AD should not be interpreted as high‐fat intake.

The AAO distribution by PGS quartiles generally shows clear differences between PGS quartiles, either increasing or decreasing AAO in the higher quartile. Both educational attainment and cardiovascular disease showed up to 1.4‐ and 1.2‐year differences between the 4^th^ and 1^st^ quartiles. This difference is even larger for LDL‐cholesterol (2.6 years) and relative fat intake (2.7 years). However, AAO differences between PGS quartiles were less for T2D, smoking behavior, age of smoking initiation, and BMI. Particularly, while T2D showed a significant causal association with AAO of AD, the usage of its PGS to predict one's AAO is less promising than the PGS for educational attainment and cardiovascular disease.

In this study, we generated two types of PGS as putative modifiable factors. We expected that PGS_Cat_ from the PGS Catalog would explain the exposure better, considering it was trained for the exposure directly. However, it is limited by the availability of published PGSs and the robustness of the original studies. On the other hand, the cross‐trait PGS_C+T_ approach was based on the variant sets achieving the best prediction (highest *R* ^2^) of AAO. One concern is whether variants selected for computing PGS_C+T_ are deemed to relate to the exposure. To address this concern, we set the variant selection p‐thresholds ranging from 0.01 to 5 × 10^−8^, instead of starting from 0.1 as in common practice, recognizing that we may still miss some variants that contribute to the overall performance of exposure‐PGS. High correlation and relatively consistent association results between PGS_C+T_ and PGS_Cat_ observed for the majority of top exposures were encouraging. However, age of smoking initiation and BMI were two exceptions with inverse correlation, and smoking behavior has a relatively lower correlation (Table S4). While we do not have a definite explanation, it is possible that the variants included in both types of PGSs may not fully capture the underlying genetic propensity of these exposures, either reflecting a substantial degree of missing heritability or exhibiting a lower signal‐to‐noise ratio. Overall, we recommend applying both approaches to validate the findings. In particular, PGS_C+T_ offers an opportunity to explore a broader range of potential risk factors (e.g., relative fat intake, cognitive performance) when published PGSs are unavailable.

Some known limitations exist in the PGS and MR analyses, but we have tried to minimize them. First, we cannot validate the results based on observed exposure data since they are unavailable in our AD genetic dataset. Therefore, in this study, we assume each exposure‐PGS is an informed proxy for the exposure. Our strategy to analyze both PGS_C+T_ and PGS_Cat_ also strengthens the confidence of the association result, as explained above. Second, less robust IVs or limited sample sizes in MR analysis can lead to biased results. To mitigate these issues, we restricted IVs to variants strongly associated with each exposure, applying two *p*‐value thresholds (*p* < 5 × 10^−8^ and *p* < 5 × 10^−6^) and requiring F‐statistics ≥10 to ensure sufficient instrument strength. The causal factors identified by the two‐sample MR analysis were further validated using the one‐sample MR method. Moreover, all GWAS summary statistics for both exposures and the AAO of AD were based on large sample sizes, reducing the risk of weak‐instrument bias. We also used several additional MR methods to address the potential horizontal pleiotropy, outliers, and reverse causality. Finally, to address the validity of our MR analysis, we conducted negative control analyses using height and rheumatologic conditions, which are not expected to be causally related to AD or its AAO; as expected, no significant associations were observed.

In summary, a higher genetic predisposition of educational attainment, cardiovascular disease, and type 2 diabetes appears to have an association and causal effect on AAO of AD, supported by both PGS and MR analysis. An additional five other PGS putative modifiable factors demonstrated a significant association with AAO of AD. These findings are corroborated by employing two different methods for generating exposure‐PGS (PGS_C+T_ and PGS_Cat_) and two analytical approaches to model AAO as both a quantitative and survival trait.

## CONFLICT OF INTEREST STATEMENT

The authors have no conflicts of interest to declare. Author disclosures are available in the Supporting Information.

## CONSENT STATEMENT

All study participants enrolled in the cohorts of the Alzheimer's Disease Genetic Consortium (ADGC) provided informed consent.

## Supporting information



Supporting Information

Supporting Information

Supporting Information

Supporting Information
