# Supplementary figures and images for: Leveraging genetic propensity to identify modifiable factors for the age at onset of Alzheimer's disease
Source: Alzheimers Dement. 2026 Feb 8;22(2):e71111. doi: 10.1002/alz.71111 (PMC12883339; doi:10.1002/alz.71111)

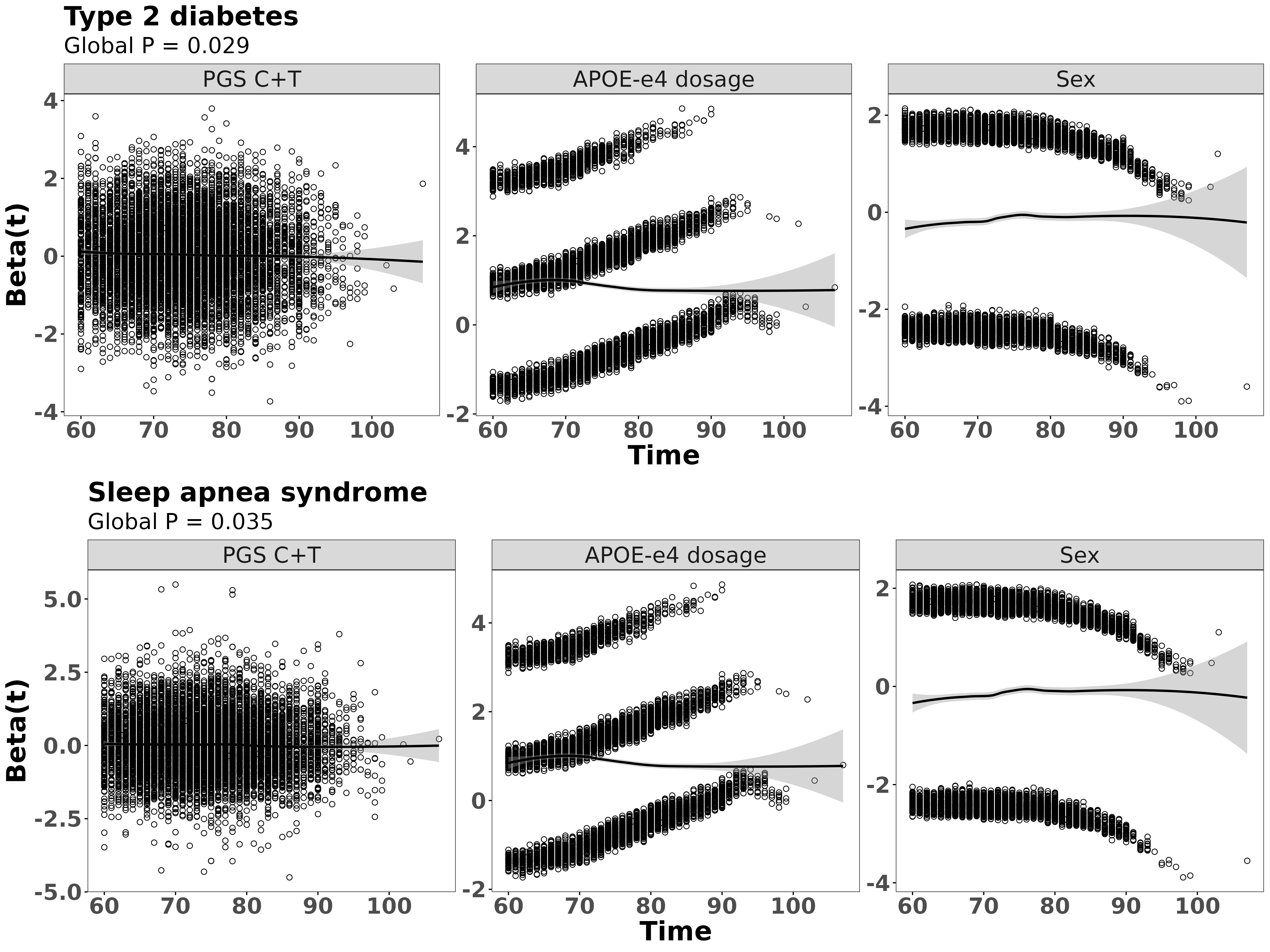

Supplement: Supplementary file 1 — Supporting Information [file ALZ-22-e71111-s001.jpeg]

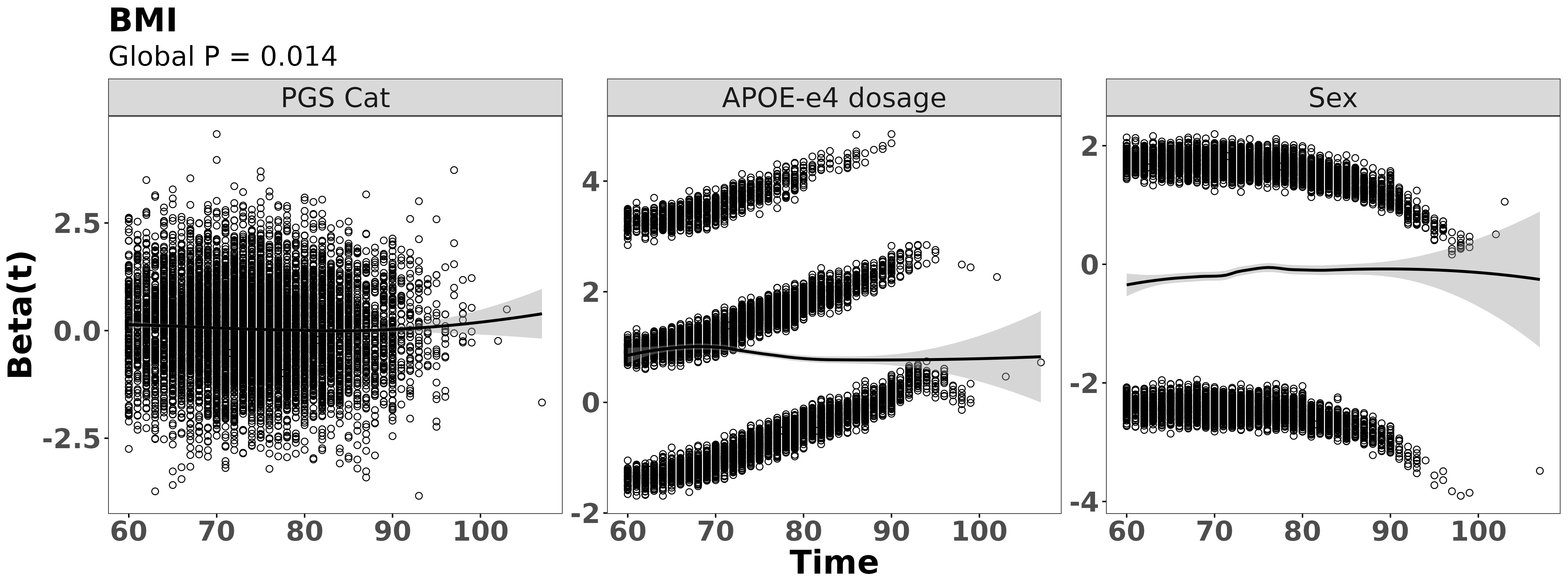

Supplement: Supplementary file 2 — Supporting Information [file ALZ-22-e71111-s003.jpeg]
