# Supplementary material for: Leveraging genetic propensity to identify modifiable factors for the age at onset of Alzheimer's disease
Source: Alzheimers Dement. 2026 Feb 8;22(2):e71111. doi: 10.1002/alz.71111 (PMC12883339; doi:10.1002/alz.71111)
Supplement: Supplementary file 3 — Supporting Information [file ALZ-22-e71111-s004.docx]

**Supplementary Information**

**Leveraging genetic propensity to identify modifiable factors for the age at onset of Alzheimer’s disease**

Yi-Ju Li^1,2,3,*^, Jong Ok La^2^, Adam Naj^4^, Eden R. Martin^5,6^

1. Department of Biostatistics and Bioinformatics, Duke University School of Medicine, Durham, NC, USA
2. Duke Molecular Physiology Institute, Duke University School of Medicine, Durham, NC, USA
3. Centre of Quantitative Medicine, Duke-NUS Medical School, Singapore
4. Department of Biostatistics, Epidemiology, and Informatics, University of Pennsylvania Perelman School of Medicine, Philadelphia, Pennsylvania, USA
5. John P. Hussman Institute for Human Genomics, University of Miami Miller School of Medicine, Miami, Florida, USA
6. John T. MacDonald Foundation Department of Human Genetics, University of Miami, Miller School of Medicine, Miami, Florida, USA

**Supplementary Figure Legends**

**Figure S1:** Schoenfeld residuals versus event time for Type 2 diabetes and sleep apnea for the main covariates adjusted in the survival model, PGS_C+T_, APOE-ε4, and sex. Global test p-value for testing the proportional hazard (PH) assumption was obtained from the cox.zph() function and presented

**Figure S2:** Schoenfeld residuals versus event time for BMI for the main covariates adjusted in the survival model, PGS_Cat_, APOE-ε4, and sex. Global test p-value for testing the proportional hazard (PH) assumption was obtained from the cox.zph() function and presented

**Supplementary Tables**

**Table S1**. Results for exposures associated with the AAO of AD. A total of 27 exposures with exposure-PGS_C+T_ meeting p < 0.05. Among them, 13 exposures with PGS from the PGS catalog were tested for association tests.

|  | **PGS from PRCise C+T method (PGC_C + T_)** | | | | **PGS Catalog (PGS_CAT_)** | | | |
| --- | --- | --- | --- | --- | --- | --- | --- | --- |
|  | **LMM** | | **CoxPHF** | | **LMM** | | **CoxPHF** | |
| **Exposure** | **Beta (95% CI)** | **P value** | **HR (95% CI)** | **P value** | **Beta (95% CI)** | **P value** | **HR (95% CI)** | **P value** |
| **Factors with exposure-PGS_C+T_ meeting p<0.05** | | | | | | | | |
| Cardiovascular disease | -0.31 (-0.44, -0.17) | <0.001 | 1.04 (1.02, 1.06) | <0.001 | -0.38 (-0.52, -0.24) | <0.001 | 1.07 (1.05, 1.09) | <0.001 |
| BMI | -0.02 (-0.16, 0.12) | 0.767 | 0.97 (0.95, 0.99) | 0.004 | -0.37 (-0.51, -0.24) | <0.001 | 1.05 (1.03, 1.07) | <0.001^†^ |
| Education attainment | 0.38 (0.24, 0.52) | <0.001 | 0.92 (0.9, 0.94) | <0.001 | 0.37 (0.23, 0.51) | <0.001 | 0.92 (0.9, 0.94) | <0.001 |
| Major coronary heart disease | -0.19 (-0.33, -0.05) | 0.006 | 1 (0.98, 1.02) | 0.906 | -0.33 (-0.47, -0.19) | <0.001 | 1.05 (1.03, 1.07) | <0.001 |
| Type 2 diabetes | -0.32 (-0.46, -0.18) | <0.001 | 1.04 (1.02, 1.06) | <0.001^†^ | -0.24 (-0.38, -0.1) | 0.001 | 1.02 (1, 1.05) | 0.023 |
| low-density lipoprotein (LDL) cholesterol | -0.19 (-0.34, -0.05) | 0.010 | 1.02 (1, 1.04) | 0.075 | -0.22 (-0.36, -0.07) | 0.003 | 1.04 (1.02, 1.06) | 0.001 |
| Total cholesterol | -0.18 (-0.32, -0.04) | 0.015 | 1.02 (1, 1.05) | 0.031 | -0.21 (-0.35, -0.06) | 0.005 | 1.04 (1.01, 1.06) | 0.001 |
| Age of smoking initiation | 0.13 (0, 0.27) | 0.055 | 0.97 (0.95, 0.99) | 0.003 | -0.17 (-0.31, -0.03) | 0.018 | 1.04 (1.01, 1.06) | 0.001 |
| Age-related hearing impairment | -0.11 (-0.25, 0.03) | 0.119 | 1.02 (1, 1.05) | 0.024 | 0.04 (-0.1, 0.18) | 0.560 | 1.01 (0.99, 1.03) | 0.211 |
| Drinks per week | -0.19 (-0.33, -0.05) | 0.009 | 1.03 (1.01, 1.06) | 0.001 | 0.03 (-0.11, 0.17) | 0.658 | 0.99 (0.97, 1.01) | 0.369 |
| Hypertension | -0.22 (-0.36, -0.09) | 0.001 | 1.01 (0.99, 1.04) | 0.184 | -0.03 (-0.16, 0.11) | 0.703 | 1.02 (1, 1.05) | 0.022 |
| Smoking behavior | -0.27 (-0.4, -0.13) | <0.001 | 1.05 (1.03, 1.08) | <0.001 | -0.02 (-0.16, 0.11) | 0.731 | 0.99 (0.97, 1.01) | 0.355 |
| Sleep apnea syndrome | -0.15 (-0.29, -0.01) | 0.032 | 1.02 (1, 1.04) | 0.089^†^ | 0.02 (-0.11, 0.16) | 0.739 | 0.99 (0.97, 1.01) | 0.342 |
| Cognitive performance | 0.27 (0.13, 0.4) | <0.001 | 0.93 (0.91, 0.95) | <0.001 |  |  |  |  |
| Diet: relative fat intake | 0.24 (0.09, 0.39) | 0.002 | 0.95 (0.93, 0.98) | <0.001 |  |  |  |  |
| Diet: relative protein intake | 0.1 (-0.04, 0.24) | 0.164 | 0.98 (0.96, 1) | 0.038 |  |  |  |  |
| Total fatty acid levels* | -0.16 (-0.29, -0.02) | 0.028 | 0.99 (0.97, 1.01) | 0.423 |  |  |  |  |
| Ventricular arrhythmia | 0.15 (0.01, 0.29) | 0.033 | 1 (0.98, 1.02) | 0.869 |  |  |  |  |
| Peripheral artery disease | -0.14 (-0.28, 0) | 0.043 | 1.02 (1, 1.04) | 0.123 |  |  |  |  |
| Mood swing | -0.14 (-0.28, 0) | 0.043 | 1.02 (1, 1.05) | 0.028 |  |  |  |  |
| Neurotic, stress-related and somatoform disorders | 0.11 (-0.03, 0.25) | 0.126 | 0.98 (0.96, 1) | 0.035 |  |  |  |  |
| Head injury | 0.11 (-0.03, 0.25) | 0.127 | 0.97 (0.95, 0.99) | 0.004 |  |  |  |  |
| Social isolation | -0.06 (-0.19, 0.08) | 0.416 | 1.02 (1, 1.05) | 0.025 |  |  |  |  |
| Air pollution: Particulate matter (pm2.5) | 0.04 (-0.11, 0.19) | 0.604 | 1.03 (1.01, 1.05) | 0.009 |  |  |  |  |
| Air pollution: Particulate matter (pm10) | -0.01 (-0.18, 0.15) | 0.858 | 1.02 (1, 1.05) | 0.020 |  |  |  |  |
| Air pollution: Nitrogen dioxide | 0.17 (0.01, 0.34) | 0.039 | 1.01 (0.99, 1.03) | 0.595 |  |  |  |  |
| Air pollution: Nitrogen oxides | -0.02 (-0.18, 0.14) | 0.797 | 1.02 (1, 1.05) | 0.035 |  |  |  |  |
| **Factors not associated with the AAO of AD, p > 0.05** | | | | |  |  |  |  |
| Systolic blood pressure | -0.09 (-0.22, 0.05) | 0.223 | 1.02 (1, 1.04) | 0.116 |  |  |  |  |
| Cigarettes per day | 0.09 (-0.06, 0.23) | 0.240 | 1.01 (0.99, 1.04) | 0.195 |  |  |  |  |
| Depression | -0.08 (-0.22, 0.06) | 0.254 | 1 (0.98, 1.03) | 0.640 |  |  |  |  |
| Moderate-vigorous physical activity | -0.07 (-0.21, 0.07) | 0.343 | 1 (0.98, 1.02) | 0.983 |  |  |  |  |
| Diastolic blood pressure | -0.06 (-0.2, 0.07) | 0.360 | 1.01 (0.99, 1.03) | 0.499 |  |  |  |  |
| Diet: relative sugar intake | 0.06 (-0.09, 0.21) | 0.427 | 1.01 (0.99, 1.03) | 0.468 |  |  |  |  |
| Diet: relative carbohydrate intake | 0.02 (-0.13, 0.17) | 0.767 | 1 (0.98, 1.02) | 0.968 |  |  |  |  |
| Sleep duration | -0.04 (-0.17, 0.1) | 0.601 | 0.99 (0.97, 1.01) | 0.431 |  |  |  |  |
| Triglycerides | -0.03 (-0.17, 0.1) | 0.624 | 0.99 (0.97, 1.01) | 0.316 |  |  |  |  |
| Insomnia | -0.03 (-0.17, 0.1) | 0.632 | 1.01 (0.99, 1.03) | 0.541 |  |  |  |  |
| Atrial fibrillation | 0.03 (-0.11, 0.17) | 0.659 | 1.01 (0.99, 1.03) | 0.433 |  |  |  |  |
| high-density lipoprotein cholesterol | 0 (-0.14, 0.14) | 0.954 | 1 (0.98, 1.02) | 0.924 |  |  |  |  |
| Heart failure | 0 (-0.14, 0.14) | 0.994 | 0.99 (0.97, 1.01) | 0.444 |  |  |  |  |
| Saturated fatty acid levels | 0.03 (-0.11, 0.17) | 0.689 | 1 (0.98, 1.02) | 0.983 |  |  |  |  |
| Polyunsaturated fatty acids levels | -0.05 (-0.19, 0.09) | 0.507 | 1 (0.97, 1.02) | 0.637 |  |  |  |  |
| Monounsaturated fatty acid levels | -0.02 (-0.16, 0.12) | 0.773 | 0.99 (0.97, 1.01) | 0.332 |  |  |  |  |

*Total fatty acid levels: Meta-GWAS summary statistics from saturated fatty acid levels, polyunsaturated fatty acid levels, and monounsaturated fatty acid levels.

†CoxPHF model for T2D and sleep apnea (PGS_C+T_ ), and BMI (PGS_Cat_) included additional adjustment of time×APOE-ε4 and time×sex interaction terms to address the violation of the PH assumption detected for both covariates.

**Table S2.** PGS data sources from the PGS Catalog for 13 modifiable factors (exposures) with exposure-PGS_C+T_ meeting p < 0.05

|  |  | **PGS Catalog** | |
| --- | --- | --- | --- |
| **Category** | **Trait** | **Study** | **Sample size (N)** |
| Cardiovascular Disease | Cardiovascular disease | Weissbrod et al. (2022)[24] | 337,488 |
|  | Hypertension | Ma et al. (2022)[27] | 361,194 |
|  | Major coronary heart disease | Mars et al. (2020)[22] | 408,458 |
| Lifestyle | Age of smoking initiation | Saunders et al. (2022) [29] | 323,386 |
|  | Drinks per week | Barr et al. (2020)[31] | 534,683 |
|  | Sleep apnea syndrome | Ma et al. (2022)[27] | 358,945 |
|  | Smoking behavior | Tanigawa et al. (2022)[30] | 68,516 |
| Metabolic | BMI | Ma et al. (2022)[27] | 354,831 |
|  | low-density lipoprotein cholesterol (LDL-cholesterol) | Ma et al. (2022)[27] | 343,621 |
|  | Total cholesterol | Ma et al. (2022)[27] | 344,278 |
| Pre-existing Conditions | Age-related hearing impairment | Cherny et al. (2020) [40] | 250,389 |
|  | Educational attainment | Privé et al. (2022)[34] | 391,124 |
|  | Type 2 diabetes | Ma et al. (2022)[27] | 360,192 |

**Table S3:**

**Table S3a**: Pairwise correlation between exposure-PGSs, presented by Person correlation coefficient and its 95% confidence interval (r (95% CI)) for cases and all subjects (cases and controls)

|  | Cardiovascular disease | Educational attainment | LDL cholesterol | Type 2 diabetes | Total cholesterol | Major coronary heart disease | Cognitive performance | Diet: relative fat intake |
| --- | --- | --- | --- | --- | --- | --- | --- | --- |
| Cardiovascular disease |  | -0.04 (-0.06, -0.02) | 0.08 (0.05, 0.10) | 0.08 (0.06, 0.10) | 0.07 (0.05, 0.09) | 0.06 (0.04, 0.08) | -0.01 (-0.03, 0.01) | -0.01 (-0.03, 0.01) |
| Educational attainment | -0.05 (-0.06, -0.03) |  | 0.01 (-0.01, 0.03) | -0.03 (-0.05, -0.01) | 0.03 (0.01, 0.05) | -0.003 (-0.02, 0.02) | **0.22** (0.20, 0.24) | 0.06 (0.04, 0.08) |
| LDL cholesterol | 0.07 (0.06, 0.09) | 0.01 (0, 0.02) |  | -0.01 (-0.03, 0.01) | **0.85** (0.84, 0.85) | 0.05 (0.03, 0.07) | -0.02 (-0.04, 0.005) | -0.16 (-0.18, -0.14) |
| Type 2  diabetes | 0.08 (0.07, 0.10) | -0.02 (-0.04, -0.01) | -0.001 (-0.02, 0.01) |  | -0.003 (-0.02, 0.02) | 0.02 (-0.003, 0.04) | -0.02 (-0.04, 0.003) | 0.07 (0.05, 0.09) |
| Total  cholesterol | 0.07 (0.06, 0.09) | 0.02 (0.004, 0.03) | **0.84** (0.84, 0.84) | 0.01 (-0.01, 0.02) |  | 0.05 (0.03, 0.07) | -0.01 (-0.03, 0.01) | -0.12 (-0.14, -0.10) |
| Major coronary heart disease | 0.06 (0.04, 0.07) | -0.01 (-0.02, 0.01) | 0.05 (0.03, 0.06) | 0.01 (0.001, 0.03) | 0.04 (0.03, 0.05) |  | 0.005 (-0.02, 0.02) | -0.01 (-0.03, 0.01) |
| Cognitive performance | -0.02 (-0.03, -0.002) | **0.22** (0.21, 0.23) | -0.01 (-0.03, 0.001) | -0.01 (-0.03, -0.001) | -0.01 (-0.02, 0.003) | 0.005 (-0.01, 0.02) |  | 0.02 (0.002, 0.04) |
| Diet: relative fat intake | -0.01 (-0.02, 0.01) | 0.06 (0.05, 0.08) | -0.13 (-0.15, -0.12) | 0.06 (0.04, 0.07) | -0.09 (-0.10, -0.08) | -0.01 (-0.02, 0.01) | 0.03 (0.02, 0.04) |  |

**Lower left:** exposure-PGS computed for cases and controls; **Upper right:** exposure-PGS computed for cases only

**Table S3b:** Jointly modeling correlated exposures (exposure-PGS) on the multivariable linear mixed model (LMM) and Cox proportional hazard model (CoxPHF), respectively, with covariate adjustment of sex, APOE-e4, and top 10 PCs

| **Exposure-PGS** | **Pearson correlation**  **(r)** | **Case-Only**  **LMM** | | **Pearson correlation**  **(r)** | **Case-Control**  **COXPHF** | |
| --- | --- | --- | --- | --- | --- | --- |
|  |  | **Beta (95% CI)** | **P value** |  | **HR (95% CI)** | **P value** |
| Total cholesterol | 0.85 (0.84, 0.85) | -0.07 (-0.33, 0.19) | 0.596 | 0.84 (0.84, 0.84) | 1.02 (0.98, 1.06) | 0.310 |
| LDL cholesterol |  | -0.13 (-0.4, 0.13) | 0.320 |  | 1 (0.97, 1.04) | 0.831 |
|  |  | | |  | | |
| Cognitive performance | 0.22 (0.20, 0.24) | 0.19 (0.05, 0.33) | 0.007 | 0.22 (0.21, 0.23) | 0.94 (0.92, 0.96) | < 0.001 |
| Education attainment |  | 0.34 (0.2, 0.48) | < 0.001 |  | 0.94 (0.92, 0.96) | < 0.001 |

**Table S4.** Pearson correlation (r) between PGS_C+T_ and PGS_CAT_ for the nine top modifiable factors with both PGS data

|  | **Number of Variants** | |  | **95% Confidence Interval** | |
| --- | --- | --- | --- | --- | --- |
| **Exposure** | **PGS_C+T_** | **PGS_CAT_** | **r** | **Lower limit** | **Upper limit** |
| Education attainment | 8478 | 950,842 | 0.429 | 0.41 | 0.45 |
| Cardiovascular disease | 937 | 1,106,422 | 0.487 | 0.47 | 0.50 |
| low-density lipoprotein cholesterol | 123 | 7,564,018 | 0.511 | 0.50 | 0.53 |
| Total cholesterol | 129 | 6,329,910 | 0.483 | 0.47 | 0.50 |
| Major coronary heart disease | 444 | 6,411,697 | 0.190 | 0.17 | 0.21 |
| Type 2 diabetes | 614 | 8,737,832 | 0.260 | 0.24 | 0.28 |
| Smoking behavior | 11,669 | 799 | 0.120 | 0.10 | 0.14 |
| Age of smoking initiation | 708 | 1,053,625 | -0.248 | -0.28 | -0.23 |
| BMI | 7,506 | 6,318,858 | -0.046 | -0.07 | -0.03 |

**Table S5.** Results of MR analysis for 11 top exposures, where instrumental variables (IVs) were selected based on p < 5x10^-8^

|  |  |  |  |  | **Pleiotropy  (Egger intercept)** | | **Heterogeneity (Q)** | **MR-PRESSO Global Test** |
| --- | --- | --- | --- | --- | --- | --- | --- | --- |
|  | **Method** | **SNPs** | **beta (SE)** | **p** | **beta (SE)** | **p** | **p** | **p** |
| Cardiovascular disease | IVW | 158 | -3.75 (1.17) | 0.001 |  |  | 0.27 | 0.26 |
|  | MR Egger | 158 | 0.34 (3.6) | 0.93 | -0.03 (0.03) | 0.23 | 0.28 |  |
|  | Weighted median | 158 | -3.42 (1.74) | 0.049 |  |  |  |  |
|  | Weighted mode | 158 | -1.29 (3.74) | 0.73 |  |  |  |  |
| Education attainment | IVW | 559 | 1.48 (0.35) | 2.85x10^-5^ |  |  | 0.41 | 0.41 |
|  | MR Egger | 559 | 1.56 (1.45) | 0.28 | -0.001 (0.02) | 0.95 | 0.40 |  |
|  | Weighted median | 559 | 1.35 (0.55) | 0.015 |  |  |  |  |
|  | Weighted mode | 559 | 1.78 (1.69) | 0.29 |  |  |  |  |
| Type 2 diabetes | IVW | 130 | -0.29 (0.14) | 0.046 |  |  | 0.89 | 0.89 |
|  | MR Egger | 130 | -0.77 (0.34) | 0.026 | 0.04 (0.02) | 0.12 | 0.91 |  |
|  | Weighted median | 130 | -0.67 (0.25) | 0.008 |  |  |  |  |
|  | Weighted mode | 130 | -0.71 (0.31) | 0.026 |  |  |  |  |
| Age of smoking initiation | IVW | 11 | -1.58 (1.98) | 0.43 |  |  | 0.41 | 0.43 |
|  | MR Egger | 11 | -13.59 (8.76) | 0.16 | 0.22 (0.16) | 0.19 | 0.50 |  |
|  | Weighted median | 11 | -2.32 (2.61) | 0.37 |  |  |  |  |
|  | Weighted mode | 11 | -3.33 (4.14) | 0.44 |  |  |  |  |
| BMI | IVW | 2588 | 0.04 (0.15) | 0.80 |  |  | 0.17 | 0.17 |
|  | MR Egger | 2588 | -0.16 (0.31) | 0.60 | 0.003 (0.005) | 0.45 | 0.17 |  |
|  | Weighted median | 2588 | -0.06 (0.25) | 0.81 |  |  |  |  |
|  | Weighted mode | 2588 | 0.16 (0.48) | 0.73 |  |  |  |  |
| Cognitive performance | IVW | 158 | 0.65 (0.42) | 0.12 |  |  | 0.90 | 0.90 |
|  | MR Egger | 158 | -1.07 (2.12) | 0.61 | 0.04 (0.04) | 0.41 | 0.90 |  |
|  | Weighted median | 158 | 0.49 (0.63) | 0.43 |  |  |  |  |
|  | Weighted mode | 158 | -0.1 (1.62) | 0.95 |  |  |  |  |
| Diet: relative fat intake | IVW | 4 | -1.01 (2.3) | 0.66 |  |  | 0.53 | 0.59 |
|  | MR Egger | 4 | -5.68 (10.04) | 0.63 | 0.11 (0.22) | 0.68 | 0.37 |  |
|  | Weighted median | 4 | -0.58 (2.79) | 0.84 |  |  |  |  |
|  | Weighted mode | 4 | -0.21 (3.47) | 0.96 |  |  |  |  |
| Low-density lipoprotein cholesterol | IVW | 85 | -0.62 (0.32) | 0.051 |  |  | 0.26 | 0.26 |
|  | MR Egger | 85 | -0.08 (0.5) | 0.87 | -0.03 (0.02) | 0.17 | 0.29 |  |
|  | Weighted median | 85 | -0.38 (0.48) | 0.43 |  |  |  |  |
|  | Weighted mode | 85 | -0.37 (0.42) | 0.38 |  |  |  |  |
| Smoking behavior | IVW | 293 | -0.35 (0.57) | 0.54 |  |  | 0.14 | 0.13 |
|  | MR Egger | 293 | 1.77 (2.49) | 0.48 | -0.03 (0.03) | 0.38 | 0.14 |  |
|  | Weighted median | 293 | -0.56 (0.86) | 0.52 |  |  |  |  |
|  | Weighted mode | 293 | -1.39 (2.32) | 0.55 |  |  |  |  |
| Total cholesterol | IVW | 105 | -0.53 (0.29) | 0.071 |  |  | 0.46 | 0.46 |
|  | MR Egger | 105 | -0.45 (0.5) | 0.37 | -0.004 (0.02) | 0.85 | 0.43 |  |
|  | Weighted median | 105 | -0.41 (0.47) | 0.38 |  |  |  |  |
|  | Weighted mode | 105 | -0.47 (0.44) | 0.29 |  |  |  |  |
| Major coronary heart disease | IVW | 6 | -0.87 (14.48) | 0.95 |  |  | 0.81 | 0.79 |
|  | MR Egger | 6 | -25.1 (32.23) | 0.48 | 0.09 (0.11) | 0.45 | 0.81 |  |
|  | Weighted median | 6 | -6.72 (17.6) | 0.70 |  |  |  |  |
|  | Weighted mode | 6 | -16.16 (22.12) | 0.50 |  |  |  |  |

**Table S6.** Reverse MR analysis for the selected exposures from the top 11 exposures showing PGS association

|  |  |  |  |  | **Pleiotropy (Egger intercept)** | | **Heterogeneity** | | |
| --- | --- | --- | --- | --- | --- | --- | --- | --- | --- |
| **Exposure** | **Method** | **SNPs** | **beta (SE)** | **p** | **beta (SE)** | **p** | | **p** |  |
| Education attainment | IVW | 4 | 0 (0.003) | 0.988 |  |  | | 0.009 |  |
|  | MR Egger | 4 | -0.009 (0.019) | 0.669 | 0.006 (0.01) | 0.662 | | 0.006 |  |
|  | Weighted median | 4 | -0.001 (0.002) | 0.714 |  |  | |  |  |
|  | Weighted mode | 4 | -0.004 (0.003) | 0.204 |  |  | |  |  |
| Smoking behavior | IVW | 7 | -0.001 (0.003) | 0.671 |  |  | | <0.001 |  |
|  | MR Egger | 7 | -0.032 (0.017) | 0.118 | 0.018 (0.01) | 0.127 | | 0.002 |  |
|  | Weighted median | 7 | -0.005 (0.002) | 0.018 |  |  | |  |  |
|  | Weighted mode | 7 | -0.006 (0.002) | 0.038 |  |  | |  |  |
| Cognitive performance | IVW | 5 | 0.003 (0.003) | 0.293 |  |  | | 0.141 |  |
|  | MR Egger | 5 | 0.003 (0.019) | 0.89 | 0 (0.01) | 0.974 | | 0.075 |  |
|  | Weighted median | 5 | 0.004 (0.004) | 0.299 |  |  | |  |  |
|  | Weighted mode | 5 | 0.006 (0.005) | 0.315 |  |  | |  |  |
| Diet: relative fat intake | IVW | 5 | -0.002 (0.003) | 0.582 |  |  | | 0.084 |  |
|  | MR Egger | 5 | -0.004 (0.018) | 0.848 | 0.001 (0.01) | 0.92 | | 0.042 |  |
|  | Weighted median | 5 | -0.005 (0.003) | 0.103 |  |  | |  |  |
|  | Weighted mode | 5 | -0.006 (0.004) | 0.203 |  |  | |  |  |
| Age of smoking initiation | IVW | 5 | 0.003 (0.003) | 0.301 |  |  | | 0.132 |  |
|  | MR Egger | 5 | 0.027 (0.028) | 0.411 | -0.015 (0.02) | 0.457 | | 0.128 |  |
|  | Weighted median | 5 | 0.006 (0.003) | 0.06 |  |  | |  |  |
|  | Weighted mode | 5 | 0.007 (0.005) | 0.207 |  |  | |  |  |

**Table S7.** Results of MR analysis for 11 top exposures, where instrumental variables (IVs) were selected based on p < 5x10^-6^

|  |  | **No. of SNPs** |  |  | **Pleiotropy  (Egger intercept)** | | **Heterogeneity (Q)** | **MR-PRESSO Global Test** |
| --- | --- | --- | --- | --- | --- | --- | --- | --- |
| **Exposure** | **Method** |  | **beta (SE)** | **p** | **beta (SE)** | **p** | **p** | **p** |
| Cardiovascular disease | IVW | 291 | -3.42 (1) | 0.001 |  |  | 0.24 | 0.24 |
|  | MR Egger | 291 | -0.63 (2.88) | 0.828 | -0.02 (0.02) | 0.3 | 0.24 |  |
|  | Weighted median | 291 | -3.11 (1.49) | 0.037 |  |  |  |  |
|  | Weighted mode | 291 | -1.61 (3.26) | 0.622 |  |  |  |  |
| Education attainment | IVW | 1082 | 1.33 (0.29) | 4.79x10^-6^ |  |  | 0.45 | 0.46 |
|  | MR Egger | 1082 | 2.7 (1.09) | 0.014 | -0.02 (0.01) | 0.19 | 0.46 |  |
|  | Weighted median | 1082 | 1.27 (0.44) | 0.004 |  |  |  |  |
|  | Weighted mode | 1082 | 1.76 (1.49) | 0.237 |  |  |  |  |
| Type 2 diabetes | IVW | 264 | -0.36 (0.12) | 0.003 |  |  | 0.83 | 0.83 |
|  | MR Egger | 264 | -0.44 (0.3) | 0.145 | 0.01 (0.02) | 0.76 | 0.81 |  |
|  | Weighted median | 264 | -0.59 (0.22) | 0.009 |  |  |  |  |
|  | Weighted mode | 264 | -0.7 (0.28) | 0.014 |  |  |  |  |
| Age of smoking initiation | IVW | 73 | -0.56 (0.94) | 0.552 |  |  | 0.38 | 0.39 |
|  | MR Egger | 73 | 0.18 (4.7) | 0.97 | -0.01 (0.07) | 0.87 | 0.35 |  |
|  | Weighted median | 73 | -0.55 (1.34) | 0.681 |  |  |  |  |
|  | Weighted mode | 73 | 0.67 (3.39) | 0.845 |  |  |  |  |
| BMI | IVW | 3308 | -0.01 (0.15) | 0.95 |  |  | 0.03 | 0.03 |
|  | MR Egger | 3308 | 0.01 (0.29) | 0.978 | 0 (0.004) | 0.94 | 0.03 |  |
|  | Weighted median | 3308 | -0.09 (0.23) | 0.706 |  |  |  |  |
|  | Weighted mode | 3308 | 0.07 (0.46) | 0.888 |  |  |  |  |
|  | Outlier corrected | 3306 | -0.01 (0.15) | 0.924 |  |  |  |  |
| Cognitive performance | IVW | 470 | 0.82 (0.29) | 0.005 |  |  | 0.44 | 0.44 |
|  | MR Egger | 470 | -1.03 (1.34) | 0.446 | 0.03 (0.02) | 0.16 | 0.46 |  |
|  | Weighted median | 470 | 0.55 (0.43) | 0.198 |  |  |  |  |
|  | Weighted mode | 470 | 2.05 (1.38) | 0.137 |  |  |  |  |
| Diet: relative fat intake | IVW | 30 | 0.84 (1.39) | 0.548 |  |  | 0.14 | 0.15 |
|  | MR Egger | 30 | -1.7 (6.33) | 0.791 | 0.04 (0.1) | 0.68 | 0.11 |  |
|  | Weighted median | 30 | -0.13 (1.87) | 0.946 |  |  |  |  |
|  | Weighted mode | 30 | -0.61 (2.72) | 0.824 |  |  |  |  |
| Low-density lipoprotein cholesterol | IVW | 126 | -0.74 (0.3) | 0.015 |  |  | 0.29 | 0.28 |
|  | MR Egger | 126 | -0.14 (0.46) | 0.769 | -0.03 (0.02) | 0.08 | 0.33 |  |
|  | Weighted median | 126 | -0.39 (0.49) | 0.424 |  |  |  |  |
|  | Weighted mode | 126 | -0.36 (0.44) | 0.412 |  |  |  |  |
| Smoking initiation | IVW | 749 | -1.19 (0.41) | 0.003 |  |  | 0.3 | 0.29 |
|  | MR Egger | 749 | 2.05 (1.7) | 0.227 | -0.03 (0.02) | 0.05 | 0.33 |  |
|  | Weighted median | 749 | -1.11 (0.62) | 0.070 |  |  |  |  |
|  | Weighted mode | 749 | -1.99 (2.11) | 0.347 |  |  |  |  |
| Total cholesterol | IVW | 155 | -0.59 (0.28) | 0.036 |  |  | 0.52 | 0.53 |
|  | MR Egger | 155 | -0.4 (0.45) | 0.381 | -0.01 (0.02) | 0.6 | 0.50 |  |
|  | Weighted median | 155 | -0.42 (0.47) | 0.371 |  |  |  |  |
|  | Weighted mode | 155 | -0.43 (0.47) | 0.365 |  |  |  |  |
| Major coronary heart disease | IVW | 33 | -7.74 (8.89) | 0.384 |  |  | 0.29 | 0.30 |
|  | MR Egger | 33 | 0.46 (27.12) | 0.987 | -0.02 (0.07) | 0.75 | 0.26 |  |
|  | Weighted median | 33 | -12.94 (12.74) | 0.310 |  |  |  |  |
|  | Weighted mode | 33 | 1.34 (21.05) | 0.949 |  |  |  |  |

**Supplementary Table 8.** Results of One-Sample MR analysis for 11 top exposures, where instrumental variables (IVs) were selected based on p < 5x10^-8^ and p < 5x10^-6^

|  | **IV p < 5x10^-8^** | | | | | **IV p < 5x10^-6^** | | | | |
| --- | --- | --- | --- | --- | --- | --- | --- | --- | --- | --- |
| **Exposure** | **No. of SNPs** | **LMM** | | **CoxPHF** | | **No. of SNPs** | **LMM** | | **CoxPHF** | |
|  |  | **beta (95% CI)** | **P** | **HR (95% CI)** | **P** |  | **beta (95% CI)** | **P** | **HR (95% CI)** | **P** |
| Cardiovascular disease | 172 | -0.27 (-0.4, -0.13) | <0.001 | 1.02 (1, 1.04) | 0.036 | 312 | -0.28 (-0.42, -0.14) | <0.001 | 1.03 (1.01, 1.06) | 0.002 |
| Education attainment | 579 | 0.29 (0.15, 0.43) | <0.001 | 0.94 (0.92, 0.96) | <0.001 | 1141 | 0.3 (0.16, 0.44) | <0.001 | 0.93 (0.91, 0.95) | <0.001 |
| Type 2 diabetes | 133 | -0.14 (-0.27, 0) | 0.053 | 1.02 (1, 1.04) | 0.13 | 269 | -0.21 (-0.35, -0.07) | 0.003 | 1.01 (0.99, 1.03) | 0.305 |
| Age of smoking initiation | 12 | -0.05 (-0.18, 0.09) | 0.524 | 1 (0.98, 1.02) | 0.898 | 106 | 0.01 (-0.13, 0.15) | 0.91 | 0.99 (0.97, 1.01) | 0.259 |
| BMI | 2732 | 0 (-0.14, 0.14) | 0.991 | 0.97 (0.95, 0.99) | 0.004 | 3513 | -0.02 (-0.16, 0.12) | 0.775 | 0.97 (0.95, 0.99) | 0.01 |
| Cognitive performance | 167 | 0.1 (-0.04, 0.23) | 0.169 | 0.95 (0.93, 0.97) | <0.001 | 515 | 0.19 (0.05, 0.33) | 0.006 | 0.94 (0.92, 0.96) | <0.001 |
| Diet: relative fat intake | 6 | 0.24 (0.09, 0.39) | 0.002 | 0.95 (0.93, 0.98) | <0.001 | 47 | 0.15 (0.01, 0.29) | 0.036 | 0.98 (0.96, 1) | 0.033 |
| low-density lipoprotein cholesterol | 116 | -0.18 (-0.33, -0.04) | 0.014 | 1.02 (1, 1.04) | 0.075 | 171 | -0.22 (-0.36, -0.07) | 0.004 | 1.02 (1, 1.05) | 0.033 |
| Smoking behavior | 302 | -0.05 (-0.19, 0.09) | 0.495 | 1.01 (0.99, 1.03) | 0.377 | 805 | -0.19 (-0.32, -0.05) | 0.008 | 1.03 (1.01, 1.05) | 0.002 |
| Total cholesterol | 129 | -0.18 (-0.32, -0.04) | 0.015 | 1.02 (1, 1.05) | 0.031 | 200 | -0.2 (-0.35, -0.06) | 0.006 | 1.02 (1, 1.04) | 0.1 |
| Major coronary heart disease event | 7 | 0.01 (-0.13, 0.14) | 0.932 | 1 (0.98, 1.02) | 0.906 | 42 | -0.03 (-0.16, 0.11) | 0.716 | 1 (0.98, 1.02) | 0.871 |
